# Gene Polymorphisms Associated with Osteoarthritis: Potential Implications for Nutrigenetics and Precision Nutrition

**DOI:** 10.3390/nu18061003

**Published:** 2026-03-21

**Authors:** Alessia Mariano, Anna Scotto d’Abusco, Sergio Ammendola

**Affiliations:** 1 Department of Biochemical Sciences, Sapienza University of Rome, P.le Aldo Moro, 5, 00185 Roma, Italy; 2 BeSSA Department of Wellbeing, Health and Environmental Sustainability, Sapienza University of Rome, 02100 Rieti, Italy; 3 Ambiotec di Sergio Ammendola, Via Appia Nord 47, 04012 Cisterna di Latina, Italy; ammendola@ambiotec.it

**Keywords:** osteoarthritis, gene polymorphisms, dietary patterns, nutrigenetics and nutrigenomics, precision nutrition

## Abstract

Osteoarthritis (OA) is the main degenerative joint disease affecting nearly 7% of world population. OA is a multifactorial pathology due to environmental, inflammatory and genetic causes. Recently, the diet and consumption of specific foods have been associated to onset and progression of OA. Dietary patterns, macronutrients, micronutrients, and bioactive compounds can influence inflammatory pathways, oxidative stress, and cartilage metabolism. These effects are mediated not only by structural support but also through the modulation of gene expression and cellular signaling pathways. The emerging fields of nutrigenomics and nutrigenetics provide a mechanistic framework to explain interindividual variability in dietary responses. Nutrigenomics investigates how nutrients influence gene expression and molecular pathways involved in OA pathophysiology, whereas nutrigenetics examines how genetic polymorphisms affect nutrient metabolism, bioavailability, and biological efficacy. This narrative review critically examines current evidence on the interaction between diet, nutraceuticals, and common non-pathological genetic variants in OA. We discuss whether specific dietary patterns exert genotype-independent effects or require personalized approaches to optimize outcomes. By integrating genetic, metabolic, and nutritional perspectives, this review aims to clarify inconsistent findings in the literature and to outline the potential of precision nutrition as a complementary strategy for OA prevention and management. The integration of these approaches enables the development of personalized nutritional strategies tailored to an individual’s genetic background, metabolic profile, and comorbid conditions such as obesity, cardiovascular disease, and diabetes.

## 1. Introduction

Osteoarthritis (OA) is a particular type of degenerative arthritis characterized by the progressive destruction of articular cartilage. Arthritis, more generally, is characterized by inflammation of the joints and can have various etiologies, including septic, genetic and inflammatory causes. Typically, OA causes pain, stiffness, loss of mobility, and deformity. Therefore, the organism can respond with vascular congestion, deformation of the underlying bone, instability of ligaments, muscle weakness, and overall functional limitation.

Investigations conducted in patients with both primary or secondary OA highlight that, except in case of genetic defects, nutrition has a key role in both the prevention and management of the disease [1]. Among these, several studies have focused on the effects of macro- and micronutrients, confirming the relevance of diet in OA management [2,3]. In this context, recent reviews have highlighted the impact of polyunsaturated fatty acids on inflammation and OA progression [1], while certain food-derived compounds can improve the systemic pathophysiological conditions associated with OA [4]. It is also well known that glucosamine and chondroitin sulfate, which are naturally present as constituent units of food-derived polymers, play a beneficial role in maintaining cartilage integrity and in reducing pain and inflammation associated with OA [5,6]. These compounds contribute to cartilage homeostasis by supporting extracellular matrix synthesis and by counteracting catabolic processes involved in cartilage degradation. Moreover, studies investigating the molecular mechanisms of nutraceuticals have demonstrated that their biological effects are mediated not only through structural support but also through the modulation of cellular signaling pathways. In particular, these food-derived compounds have been shown to target gene expression directly or indirectly, influencing the regulation of inflammatory mediators, matrix-degrading enzymes, and anabolic factors involved in cartilage metabolism [7].

Diet and the intake of specific food components are therefore considered modifiable factors capable of improving the course of the disease. They are known to modulate the body’s response to pathological stress by inducing changes in gene expression, a concept referred to as nutrigenomics [8]. Through this mechanism, bioactive food components interact with molecular pathways involved in inflammation, oxidative stress, and tissue metabolism, thereby influencing disease susceptibility and progression [9]. Conversely, individual genetic variability plays a crucial role in determining the body’s response to specific nutrients, a field known as nutrigenetics, which focuses on the influence of genetic polymorphisms on nutrient absorption, metabolism, and biological efficacy [10].

The integration of nutrigenomics and nutrigenetics has paved the way for the development of personalized nutrition strategies, in which dietary interventions are tailored according to an individual’s genetic background, metabolic profile, and disease risk. Such an approach holds significant promise for optimizing nutritional interventions in the prevention and management of chronic diseases, including OA.

In this scenario, common (non-pathological) genetic variants in several genes are of interest to clarify whether certain nutrients or dietary patterns act independently of genotype, or whether personalized nutritional strategies are required [11]. For instance, in this narrative review, we compile and critically discuss evidence on how diet and specific nutraceuticals may modulate the risk of developing OA or the progression of the disease in individuals carrying particular genetic polymorphisms and affected by metabolic comorbidities such as obesity, cardiovascular diseases, or diabetes. Regarding the obesity, it is a risk factor for OA onset, due to the increase in body weight, which increases the mechanical load on the articular joints. Moreover, the inflammatory condition induced by obesity further contributes to the OA onset. It is of great interest to understand whether polymorphisms present in genes not closely linked to osteoarthritis, can also have effects on the progression of obesity. The analysed studies suggest that genetic variability significantly influences the biological efficacy of certain dietary patterns and nutraceutical interventions, offering a plausible explanation for the inconsistent and sometimes contradictory results reported across the literature.

**Figure 1:**
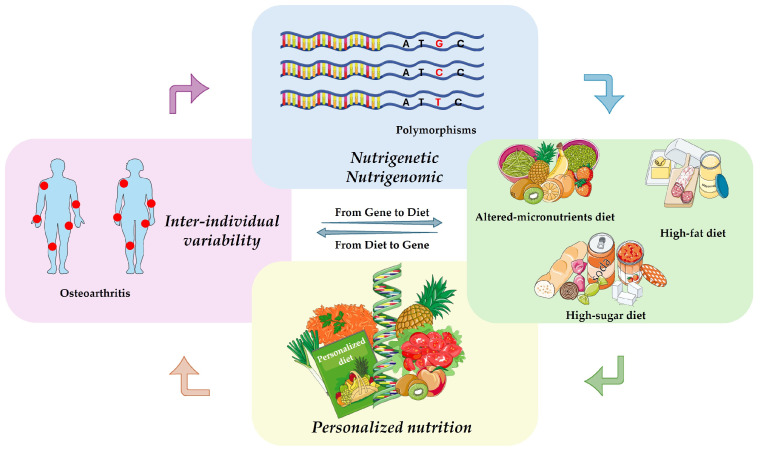
A schematic representation of the topic treated throughout the review. Images were provided by Servier Medical Art (https://smart.servier.com, accessed on 12 February 2026), licensed under CC BY 4.0.

Throughout this review, the term “diet” refers to overall eating habits rather than isolated nutrients. Accordingly, dietary patterns are categorized based on their predominant composition, including diets rich in carbohydrates, fats, micronutrients, as well as those characterized by the intake of specific bioactive nutrients. Importantly, these dietary patterns are discussed in relation to nutrient intakes that are either higher or lower than those typically associated with the Mediterranean diet, which is widely regarded as a reference model for healthy nutrition. By integrating genetic, metabolic, and nutritional perspectives, this review aims to provide a comprehensive framework for understanding interindividual variability in dietary responses and to highlight the potential of personalized nutrition strategies in the prevention and management of OA. A schematic representation of our narrative review is reported in Figure 1.

## 2. Gene Polymorphisms and Onset and Progression of OA

In the last years, an increasing number of publications have identified cellular and molecular mechanisms that contribute to the onset and progression of OA. Among these, the pro-inflammatory factors such as cytokines and chemokines as well as components of Extra-Cellular Matrix (ECM) and enzymes involved in the ECM homeostasis have been studied [12,13,14,15]. All these factors are produced by both joint cells, such as synoviocytes, chondrocytes and osteoblasts, and immune cells, such as macrophages and lymphocytes [16,17,18,19].

Growing evidence suggests that the homeostasis of these biological markers involved in OA processes can be significantly influenced by dietary habits. In particular, bioactive compounds present in foods have been shown to be associated with OA susceptibility and disease progression. These findings further support the concept that diet-gene interactions play a relevant role in modulating OA-related pathways and the adherence to a diet rich in anti-inflammatory and antioxidant compounds should be generally recommended [7].

Nutrigenetics highlights individual genetic variability that can play crucial roles in determining the response of individuals to specific nutrients. In particular, nutrigenomics studies the influence of genetic polymorphisms on nutrient absorption, metabolism, and biological efficacy. During last years, more than 300 genomic loci have been associated with OA affecting different joints. Among these loci, the vitamin D receptor (VDR) polymorphisms have been found to be very important for their role in several disorders such as OA [20]. More recently, additional 26 polymorphic genes associated with site-specific OA have been identified [21,22].

Polymorphisms in some genes may explain interindividual variability in response to nutraceutical interventions. Therefore, it seems clear that genetic variability can significantly influence the biological efficacy of certain dietary patterns and nutraceutical interventions on OA patients, and this concept can be well inserted in the personalized nutritional field [23,24]. To further complicate the picture, a brief mention of miRNA is in order, these RNAs also play an important role in gene modulation. In this review, we focus our attention on the polymorphisms of some of these 26 genes, paying special attention to the effects of the type of diet, high in fat or sugar or micronutrients in OA patients with respect to the polymorphism these patients show. The link between polymorphisms in the discussed genes and diet has not always been demonstrated. However, there are some indications that such a link may exist, for this reason, this review is also intended to stimulate further studies.

## 3. Polymorphic Genes Affected by High-Sugar Diet in OA Patients

A diet rich in carbohydrates, particularly those with a high content of simple sugars (mainly hexose ones), has a profound impact on blood glucose homeostasis, promoting insulin resistance and contributing to the development of metabolic syndrome and diabetes [25,26]. These metabolic alterations are characterized by a chronic low-grade inflammatory state, which represents a significant risk factor for the onset and progression of OA. In OA patients, sustained hyperglycemia and increased insulin signaling dysregulation enhance the production of pro-inflammatory mediators, thereby exacerbating cartilage degradation and joint inflammation [27,28]. Within this context, several genes known to exhibit polymorphic variants play a critical role in modulating inflammatory responses, metabolic pathways, and cartilage homeostasis. Importantly, the expression and activity of these genes can be influenced by dietary carbohydrate intake, particularly high-sugar diets, potentially amplifying their pathological effects in genetically predisposed individuals (Table 1). In line with the aim of the present review, we highlight key polymorphic genes whose expression is affected by dietary carbohydrates and discuss their contribution to OA susceptibility and disease progression.

### 3.1. High Mobility Group Nucleosome Binding Domain 1 (HMGN1)

HMGN1 is a chromatin-associated protein that has been shown to be dispensable for both myogenesis and adipogenesis [34]; however, it plays a relevant role as an alarmin involved in low-grade chronic inflammation, a condition strongly associated with obesity and diabetes. HMGN1 is functionally linked to High-Mobility Group Box 1 (HMGB1), a pro-inflammatory mediator whose circulating levels are elevated in diabetic individuals [35,36]. Notably, increased expression of both HMGN1 and Toll-like receptor 4 (TLR4) has been reported in diabetic kidney damage, supporting their involvement in glucose-driven inflammatory pathways [37]. HMGN1 can also bind the Receptor for Advanced Glycation End Products (RAGE), thereby activating inflammatory signaling cascades. The interaction between HMGN1 and HMGB1 is particularly relevant in the context of diets rich in simple or complex carbohydrates, which enhance the formation of AGEs and represent a recognized risk factor for OA progression. In cellular models, HMGB1 expression and activity have been shown to be modulated by glucose levels through the NAD-dependent deacetylase sirtuin-1 (SIRT1), highlighting a mechanistic link between glucose metabolism and inflammatory regulation [35]. Furthermore, SIRT1 signaling pathway represents a link between hyperhomocysteinemia (HHcy) and osteoarthritis. HHcy is very common in subjects showing polymorphism of the gene encoding Methylene Tetra Hydro Folate Reductase (MTHFR). To best of our knowledge, variant C677T (rs1801133) has been reported to be associated with primary knee osteoarthritis only in a South Indian population [38,39]. Collectively, these findings suggest that HMGN1 may contribute to OA progression in individuals with high carbohydrate and simple sugar dietary habits. Consistently, OA progression, particularly affecting the knee and hip joints, appears to be accelerated in individuals carrying the rs9981884 polymorphism in the HMGN1 gene, suggesting a genetic predisposition to enhanced inflammatory and metabolic dysregulation within joint tissues [29]. This variant may exacerbate the pro-inflammatory effects of a high-carbohydrate diet by modulating HMGN1 expression or activity, thereby amplifying AGE–RAGE signaling and HMGB1-associated inflammatory pathways. Such mechanisms provide further support for a gene–diet interaction in OA susceptibility, whereby dietary sugar intake may differentially influence disease progression in genetically predisposed individuals.

### 3.2. Pappalysin 1 (PAPP-A)

Pregnancy-associated plasma protein-A (PAPP-A), also called pappalysin, is a secreted metalloproteinase that cleaves members of the insulin-like growth factor binding protein (IGFBP) family, thereby increasing the local bioavailability of insulin-like growth factors (IGFs). PAPP-A is expressed in osteoblasts, and its enzymatic activity has been proposed to contribute to OA progression through the release of bioactive IGF within cartilage tissue, potentially affecting chondrocyte metabolism and matrix turnover [40]. Beyond joint biology, circulating PAPP-A levels have been identified as a significant predictor of obesity risk during the third trimester of pregnancy, suggesting a broader involvement of this protein in metabolic regulation [41]. However, to date, no scientifically validated evidence supports a direct association between dietary patterns, specific nutrients, or carbohydrate intake and PAPP-A expression levels. In this context, genetic variability appears to be the primary determinant of PAPP-A–related OA susceptibility. Notably, the rs1321917 polymorphism in the PAPP-A gene has been associated with an increased risk of hip OA and with reduced glomerular filtration rate, indicating a potential shared genetic background linking joint degeneration and metabolic or renal dysfunction [30]. These observations highlight PAPP-A polymorphisms as relevant contributors to OA risk, independent of direct dietary modulation, yet potentially interacting with systemic metabolic conditions that characterize OA patients. On the other hand, the miRNA-656-3p targets PAPP-A and it has been observed that circular form of pappalysin-1 mRNA enhances glycolysis via miRNA-656-3p in patients with colon cancer [42].

### 3.3. Bone Morphogenetic Protein 6 (BMP-6)

BMP-6 plays a complex and context-dependent role in metabolic disorders and skeletal homeostasis. In diabetes, reduced BMP-6 levels have been associated with bone loss in type 1 diabetes (T1DM), whereas elevated BMP-6 concentrations have been linked to cardiovascular complications in diabetic patients. Experimental evidence indicates that increasing BMP-6 expression can improve bone density in T1DM patients and contribute to glucose metabolism regulation in type 2 diabetes (T2DM), highlighting BMP-6 as a potential therapeutic target for both diabetes-associated bone impairment and metabolic control [43]. Despite these findings, there is currently no solid evidence supporting a direct association between specific dietary components and BMP-6 expression, although ascorbic acid has been reported to upregulate BMP-6 or enhance its downstream effects in bone cells [44]. Dietary patterns may nevertheless influence BMP-6 indirectly. High-fat diets (HFDs) have been shown to modulate BMP-6 levels in a tissue- and context-dependent manner, particularly in the liver [45]. Consistently, in a murine model of metabolic dysfunction-associated steatotic liver disease (MASLD), BMP-6 expression was upregulated and correlated with hepatic steatosis, but not with liver inflammation [46]. Emerging evidence also suggests an interplay between BMP-6, serotonin signaling, and glucose metabolism, with opposing effects on insulin secretion [47]. In this framework, diets rich in tryptophan, an amino acid precursor of serotonin, may indirectly influence BMP-6 activity through serotonin-mediated pathways, potentially contributing to inflammatory processes and increasing OA risk [48]. Additionally, post-transcriptional regulation further modulates BMP-6 function, as miRNA-451a has been shown to target BMP6, inhibiting its activity in bone tissue [49]. Importantly, genetic variability appears to be a critical determinant of BMP-6–related OA susceptibility. The rs11243284 and rs270417 polymorphisms in the BMP6 gene have been associated with erosive hand and hip OA, underscoring the relevance of gene–metabolism interactions in disease progression and suggesting that dietary influences may exert differential effects in genetically predisposed individuals [31].

### 3.4. Cell Migration Inducing Hyaluronidase 1 (CEMIP)

Genetic variability in the CEMIP gene appears to be a relevant determinant of OA susceptibility, particularly in metabolically compromised individuals. Notably, the rs117564279 polymorphism in CEMIP has been associated with an increased risk of hip OA characterized by pronounced cartilage loss, highlighting a genetically driven vulnerability of hyaluronan turnover within joint tissues [32]. CEMIP catalyses the catabolism of hyaluronan and participates in the regulation of hyaluronic acid–rich organs. In chondrocytes, its expression is strongly induced by inflammatory cytokines, linking CEMIP activity to inflammation-mediated cartilage degradation [50]. Beyond cartilage, CEMIP indirectly affects the integrity of the endothelial glycocalyx (GCX), a specialized extracellular matrix composed of proteoglycans, glycosaminoglycans, and glycoproteins that covers the apical surface of various cell types. Alterations in GCX thickness and permeability are characteristic of metabolic disorders, particularly diabetes, and contribute to vascular dysfunction [51]. Diet-induced metabolic imbalance further modulates this axis: Western diets rich in sugars have been shown to induce glycocalyx thickening, which in turn affects inflammation, cell adhesion, and matrix homeostasis [52]. Conversely, glycocalyx degradation, often associated with dysregulated sugar metabolism, exposes joint cell matrices to hyaluronidases, promoting syndecan degradation and accelerating cartilage degeneration [53]. High-glucose dietary patterns may also increase CEMIP expression indirectly by fostering obesity and insulin resistance, conditions in which CEMIP plays a regulatory role in adipogenesis and energy metabolism. In human studies, CEMIP expression in adipose tissue positively correlates with insulin resistance, dyslipidemia, and fasting glucose levels, and has been shown to modulate osteopontin–integrin interactions and AKT/ERK signaling pathways, supporting its role in daily energy expenditure and caloric balance [54]. Post-transcriptional regulation adds an additional layer of complexity, as CEMIP is targeted by miRNA-148a-3p, a microRNA abundant in milk, potentially influencing its expression in response to dietary patterns. Taken together, these findings suggest that CEMIP polymorphisms may amplify the detrimental effects of metabolic and dietary stressors on joint tissues, thereby contributing to OA onset and progression in genetically predisposed individuals.

### 3.5. Transforming Acidic Coiled-Coil Containing Protein 3 (TACC3)

Genetic variation in the TACC3 gene has emerged as a relevant factor in OA susceptibility, particularly in knee OA. Specifically, the rs7680647 and rs4865462 polymorphisms in TACC3 have been associated with an increased risk of OA, suggesting that altered regulation of this gene may contribute to joint degeneration in genetically predisposed individuals [22]. TACC3 is a critical regulator of chromosome alignment during mitosis and is essential for cellular survival. Proper TACC3 expression is required for chondrocyte differentiation, supporting its role in cartilage development and maintenance [55], whereas reduced TACC3 levels are associated with mitotic defects and cellular instability [56]. Conversely, pathological conditions characterized by cellular stress and necrosis can induce inflammatory responses that lead to TACC3 overexpression. In this context, elevated TACC3 levels have been shown to enhance glycolytic flux, increasing glucose consumption and lactate production, thereby promoting disease progression in highly proliferative tissues [57]. Although direct evidence linking specific dietary components to TACC3 expression is currently lacking, excessive intake of simple sugars may exacerbate glycolysis-driven metabolic stress in tissues where TACC3 is dysregulated. Taken together, these observations suggest that TACC3 polymorphisms may sensitize joint tissues to metabolic and inflammatory imbalances, potentially accelerating OA development, particularly in the knee joint.

### 3.6. Ubiquitin-Specific Peptidase 8 (USP8)

Genetic variation in the USP8 gene appears to contribute to OA susceptibility, particularly in knee OA. The rs4380013 polymorphism within the USP8 gene has been associated with an increased risk of OA onset, suggesting that altered regulation of deubiquitination pathways may predispose joint tissues to degenerative processes [33]. USP8 is a deubiquitinating enzyme with pleiotropic functions, playing a key role in protein degradation pathways that regulate multiple cellular processes, including the differentiation of osteoprogenitors into mature osteoblasts [58,59]. Consistently, USP8 expression and activity have been proposed as critical modulators of OA progression, acting as regulators of inflammatory signaling and extracellular matrix breakdown within joint tissues [60]. Beyond skeletal homeostasis, USP8 polymorphisms have been indirectly linked to metabolic disorders such as diabetes through their association with the leptin receptor Rb (LR-b), a key regulator of glucose homeostasis and energy balance [61,62]. Post-transcriptional mechanisms further modulate USP8 function, as miRNA-874-3p targets the USP8 transcript and has been shown to exert anabolic effects on skeletogenesis and bone mineralization [63,64]. Notably, miRNA-874-3p expression is influenced by glucose metabolism and by bioactive compounds with anti-inflammatory properties, such as glycyrol from Glycyrrhiza glabra and polyphenols present in certain teas, which have been explored as adjunctive agents in OA management [65,66]. Although direct dietary regulation of USP8 has not been conclusively demonstrated, these metabolic interactions suggest that nutritional factors may indirectly influence USP8-related pathways. Taken together, these findings support a role for USP8 polymorphisms in modulating OA risk, potentially amplifying the impact of metabolic and inflammatory stressors in genetically predisposed individuals.

## 4. Polymorphic Genes Affected by High-Fat Diet in OA Patients

People with an excess of fat mass relative to lean mass exhibit chronic low-grade inflammation. This condition is often exacerbated by weight gain which, by increasing mechanical load on the joints, accelerates cartilage wear and degeneration. Therefore, in addition to consuming anti-inflammatory nutrients, generally abundant in vegetables, these individuals should adopt appropriate caloric restriction and increase physical activity. Furthermore, a percentage of fats, predominantly unsaturated, should be consumed, in amount of no lower than 12% of the caloric sources, in accordance with the principles of the Mediterranean diet. These concentrations are thought to be not pro-inflammatory and generally do not result in malabsorption of essential fats [67].

It is important to note that certain foods can directly or indirectly modulate gene expression, thereby promoting obesity and other diseases, including OA. Moreover, OA progression, as said above, is influenced by gene polymorphisms which, by affecting gene expression levels, alter cellular metabolism and consequently the body’s response to dietary intake. Below, we report several genes whose polymorphisms are associated with OA and whose expression may be directly or indirectly influenced by diet through miRNA-mediated gene targeting (Table 2).

### 4.1. Collagen Beta(1-O)-Galactosyltransferase 2 (COLGALT2)

COLGALT1 polymorphisms and mutations, particularly biallelic variants, are implicated in rare, severe connective tissue diseases [70]. The gene plays a critical role in type IV collagen maturation, with dysfunction impacting ECM stability, collagen secretion, and cell adhesion. Indeed, COLGALT2 encodes an enzyme involved in collagen post-translational modification, specifically catalyzing the initial galactosylation of hydroxylysine residues in the endoplasmic reticulum [71].

It has been demonstrated that OA patients carrying specific COLGALT2 polymorphisms (rs11583641, rs1046934, rs12047271, and rs1327123) exhibit an elevated hip OA risk by inducing enhancer hypomethylation in chondrocytes. This hypomethylation leads to overexpression of COLGALT2 in cartilage cells [72].

On the other hand, its overexpression COLGALT2 has been linked to increased lipid accumulation, while, COLGALT2 deficiency has been associated with altered lipid metabolism and reduced secretion of high-molecular-weight (HMW) adiponectin [73]. Given the close interplay between lipid metabolism, low-grade inflammation, and OA progression, diet-induced modulation of COLGALT2 expression may represent a mechanistic link between metabolic status and joint degeneration [73]. In addition, COLGALT2 expression is upregulated by miRNA-135a-5p. In knee OA, statistical analyses have shown that this miRNA is associated with an increased risk of disease [68,72,74,75]. This interesting link therefore suggests that personalized nutritional interventions, particularly those targeting dietary fat composition, may represent a supportive approach to mitigate disease risk and progression in genetically predisposed individuals.

### 4.2. Metastasis Associated Lung Adenocarcinoma Transcript 1 (MALAT1)

MALAT1 is a long non-coding RNA that is now considered a biomarker and potential therapeutic target in almost all solid tumors. However, it has multiple roles. It is involved in the regulation of adipogenesis and fat deposition, influencing pathways as PPARγ and it has been shown to exert protective effects against osteoporosis and to promote cartilage homeostasis [69,76,77]. Several MALAT1 polymorphisms have been identified, such as rs3200401, rs619586, rs664589, rs600231, rs4102217, and rs10896015, described as associated with different kind of diseases, from cancer to inflammatory conditions, such as OA [78,79]. In particular, carriers of the rs10896015 polymorphism exhibit elevated serum alkaline phosphatase levels and a significantly increased risk of hip OA, suggesting a potential role for MALAT1 in bone and cartilage remodeling processes [69]. Given the regulatory functions of MALAT1 in joint tissue signaling, such polymorphisms may contribute to interindividual differences in disease onset and progression by modulating gene expression and downstream molecular pathways.

Attention must be paid to the carriers of these polymorphisms, since dietary factors appear to further modulate MALAT1 expression, and thus can influence the progression of the pathology. High-fat diets and certain lipotoxic compounds, such as palmitic acid, commonly found in animal-derived foods and some vegetable oils, have been shown to increase MALAT1 levels [80], contributing to alterate the cartilage homeostasis in OA patients. In contrast, quercetin, a plant-derived flavonoid that typically shows anti-inflammatory properties, has been reported to reduce MALAT1 expression [81], making it suitable as a supplement in nutrition in patients with OA and MALAT1 polymorphisms.

MALAT1, together with miRNA-155 and miRNA-181a, is involved in multiple physiological and pathological processes, including immune regulation and food allergy responses [82,83]. These findings support the relevance of MALAT1 genetic variants as biomarkers for OA risk stratification and as potential targets for personalized nutritional strategies.

### 4.3. Opsin 1, Short Wave Sensitive (OPN1SW)

The OPN1SW gene encodes the short-wavelength–sensitive (blue) cone photopigment and is primarily expressed in the retina [84]. Expression of OPN1SW has been shown to be sensitive to hyperoxic conditions, suggesting a vulnerability to oxidative stress–related mechanisms [85]. Under conditions of oxidative stress, activated platelets can release circulating pro-inflammatory mediators that contribute to systemic inflammation and may indirectly affect ECM homeostasis, in peripheral tissues, including articular cartilage [86]. Such systemic effects provide a plausible mechanistic link between OPN1SW dysregulation and joint tissue remodeling, potentially explaining the increased risk of hip OA observed in individuals carrying the OPN1SW polymorphism rs62479589 [32].

Dietary patterns characterized by high fat intake and excess caloric consumption are known to promote oxidative stress and chronic low-grade inflammation [87], which may exacerbate the pathogenic consequences of OPN1SW genetic variants. Although no specific foods or dietary components have been directly shown to regulate OPN1SW expression or activity, metabolic stress induced by unfavorable dietary habits may exacerbate oxidative and inflammatory pathways relevant to OA development. Taken together, these findings suggest that individuals harboring OPN1SW risk polymorphisms could benefit from personalized nutritional strategies aimed at reducing caloric excess and dietary fat intake, thereby mitigating systemic oxidative stress and potentially lowering OA risk. microRNAs represent an additional layer of gene–environment interaction. Several light-responsive microRNAs expressed in the retina have been identified as regulators of OPN1SW, among which the miRNA-204 family is upregulated in response to blue-light exposure [88]. Notably, miRNA-204 is also implicated in osteoarthritis pathophysiology, as its mature form is upregulated in senescent chondrocytes and promotes OA progression [89]. Furthermore, miRNA-204-3p expression has been reported to increase in response to high-fat diets and excess caloric intake [32,90].

### 4.4. RNA-Binding Protein 5 (RBM5)

RBM5 is an RNA-binding protein involved in the regulation of several cellular processes, including cell proliferation and alternative splicing [91,92]. Elevated expression of RBM5 has been observed in patients with osteoporosis, suggesting a potential indirect effect on osteoclast differentiation and bone remodeling [63]. Importantly, genetic variation within the RBM5 gene may modulate OA susceptibility. Specifically, individuals carrying the rs62262139 polymorphism exhibit an increased risk of developing OA at various joint sites [22].

Environmental and lifestyle factors, particularly dietary composition, may interact with these genetic variants to influence disease risk. Diets high in saturated fat, associated with systemic inflammation and oxidative stress pathways, could exacerbate RBM5-mediated alterations in cartilage and bone tissue. Conversely, a low-fat diet may reduce metabolic stress and inflammatory signaling, potentially mitigating the impact of risk-associated RBM5 polymorphisms on OA progression. These observations highlight the potential value of personalized nutritional strategies in individuals carrying high-risk RBM5 alleles, emphasizing the importance of moderating dietary fat intake to support joint health [93]. miRNA-938 is upregulated in the time interval between fasting and after ingestion of a meal high in saturated fat [94].

### 4.5. Sprouty RTK Signalling Antagonist 4 (SPRY4)

SPRY4 is an inhibitor of the receptor-transduced mitogen-activated protein kinase (MAPK) signaling pathway [95] and is considered an indicator of OA severity [96]. SPRY4 promotes adipogenesis, an inflammatory process contributing to OA, suggesting that a low-fat diet may help limit its harmful effects [97]. Excess SPRY4 can damage cartilage and increase the risk of other metabolic disorders, such as diabetes and kidney failure [95].

miRNA-92a inhibits SPRY4 expression, and butyric acid, produced by certain gut microorganisms, can suppress miRNA-92a [98]. In obesity, elevated circulating miRNA-92a levels are associated with impaired glucose metabolism [99]. Together with a high-fat diet, these conditions may accelerate OA onset and progression.

Importantly, its genetic polymorphisms can influence individual susceptibility and dietary recommendations. Carriers of the rs10062749 polymorphism in the SPRY4 gene are at increased risk for developing hand OA, whereas carriers of rs10038860 in the SPRY4-AS1 transcript have a higher risk of knee OA [30,33]. These findings suggest that individuals with these polymorphisms may particularly benefit from a low-fat diet, which could help mitigate SPRY4-related metabolic and inflammatory effects, thereby reducing OA risk and progression.

## 5. Polymorphic Genes Affected by Altered Micronutrients Diet in OA Patients

Micronutrients play a crucial role in cellular metabolism, mainly acting as cofactors or coenzymes in essential biochemical reactions; therefore, both their deficiency and excess can significantly affect cell viability and function. Vitamins and minerals, or their dietary precursors, are obtained through nutrition and subsequently converted into their biologically active forms. Variations in micronutrient availability, together with genetic background, may influence metabolic pathways involved in joint homeostasis. In this context, several genes encoding micronutrient-dependent proteins have been identified as susceptible to polymorphisms that may contribute to OA onset and progression, particularly in the presence of a low-micronutrient diet (Table 3).

### 5.1. Cyclin-Dependent Kinase 2-Associated Protein 1 (CDK2AP1)

CDK2-associated protein 1 (CDK2AP1) is a specific inhibitor of Cyclin-Dependent Kinase 2 (CDK2), a key regulator of cell cycle progression and pancreatic islet β-cell proliferation [103]. CDK2 activity is essential for the G1/S transition, DNA replication, and progression through the G2 phase; its downregulation leads to G1/S and S/G2 cell cycle arrest [104]. In type 2 diabetes, reduced CDK2 levels in pancreatic islets impair insulin secretion, highlighting the metabolic relevance of this pathway.

CDK2 expression and activity are strongly influenced by micronutrient-related mechanisms. Retinoic acid, the active form of vitamin A, modulates CDK2 expression and can induce cell cycle arrest, suggesting that dietary intake of vitamin A and carotenoids may directly affect cell cycle regulation [105]. In addition, dietary restriction has been shown to modulate CDK2AP1 expression, indirectly influencing CDK2-regulated cell cycle genes [106]. Importantly, CDK2AP1 is also regulated at the post-transcriptional level by miRNA-21, a pro-inflammatory microRNA that downregulates CDK2AP1 expression [107]. Several dietary components, including monounsaturated fatty acids (MUFA) and butyric acid, have been shown to increase miRNA-21 expression, thereby potentially reducing CDK2AP1 activity and altering cell cycle control [108,109]. Moreover, cow’s milk contains high levels of miRNA-21, further contributing to the modulation of CDK2AP1 expression through dietary exposure [110].

The relevance of these mechanisms becomes particularly evident in the context of genetic variability. Individuals carrying the CDK2AP1 polymorphism rs1060105 show an increased susceptibility to the effects of specific dietary patterns. In these subjects, diets rich in MUFA or cow’s milk and dairy products have been associated with a higher risk of knee osteoarthritis development [100]. These findings underscore the importance of considering CDK2AP1 genetic polymorphisms when defining dietary recommendations, as gene–diet interactions may significantly influence cell cycle regulation, inflammatory responses, and OA risk.

### 5.2. Secretory Carrier Membrane Protein 2 (SCAMP2)

This protein plays a key role in the regulation of T-type calcium channel trafficking and is involved in molecular transport within neuronal, endocrine, and exocrine cells. T-type voltage-sensitive calcium channels (T-VSCCs) are critically involved in the progression of OA, as they mediate nociceptive pain signaling and mechanotransduction within the joint. Among them, the T-type calcium channel CaV3.2 is highly expressed in primary sensory neurons and has been identified as the main contributor to joint pain perception [111].

Beyond pain signaling, CaV3.2 also plays a role in metabolic regulation. Recent evidence suggests that CaV3.2 is involved in hunger modulation and obesity-related pathways [112]. This dual role provides a strong rationale for dietary interventions in OA patients. In particular, low-sodium and hypocaloric diets may be especially beneficial, not only for reducing mechanical load on joints but also for modulating calcium channel activity and systemic inflammation.

Importantly, genetic variability further modulates these mechanisms. Individuals carrying the rs35206230 polymorphism in the SCAMP2 gene show an increased susceptibility to OA across multiple anatomical sites, with a particular predisposition to knee and hip involvement [113]. Since SCAMP2 is involved in vesicular trafficking and calcium channel regulation, this polymorphism may influence CaV3.2 function and, consequently, pain perception and metabolic responses [101]. Taken together, these findings highlight the importance of genetic polymorphisms in shaping both disease progression and therapeutic strategies in OA. Incorporating genetic information, such as SCAMP2 variants, into dietary planning could support a more personalized nutritional approach, optimizing pain management, metabolic control, and overall disease outcomes in OA patients.

### 5.3. Solute Carrier Family 44 Member 2 (SLC44A2)

SLC44A2 encodes choline transporter-like protein 2 (CTL2), a transmembrane protein responsible for choline and ethanolamine transport, with critical functions in the inner ear, mitochondrial metabolism, and platelet activation. Dysregulation of SLC44A2 expression has been linked to pathological phenotypic switching of vascular smooth muscle cells (VSMCs) from a quiescent, contractile state to a proliferative and synthetic phenotype, thereby contributing to vascular disease progression. In particular, elevated SLC44A2 levels have been implicated in aortic aneurysm development and venous thromboembolism (VTE) [114]. Recent evidence also highlights a role for SLC44A2 in OA, where increased protein levels or functional alterations have been observed in affected patients [115]. Importantly, genetic variability within the SLC44A2 gene appears to modulate disease susceptibility. Polymorphisms in SLC44A2 are associated with an increased risk of VTE, likely through altered platelet activation and mitochondrial dysfunction that favor thrombus formation. Moreover, carriers of the rs10405617 polymorphism show a significantly increased risk of developing OA, further supporting a shared molecular pathway linking vascular dysfunction, inflammation, and joint degeneration [30].

From a nutritional perspective, choline represents a key micronutrient with direct relevance to SLC44A2 function. Choline serves as a major source of methyl groups and is essential for the biosynthesis of phosphatidylcholine and sphingomyelin, two fundamental phospholipids required for membrane integrity, mitochondrial function, and cellular signaling. Emerging evidence suggests that diets rich in choline may downregulate SLC44A2 expression, as observed during fetal development, potentially through epigenetic and feedback-regulatory mechanisms [116]. Taken together, these findings emphasize the importance of SLC44A2 genetic polymorphisms in determining individual susceptibility to both OA and thrombotic disorders. They also support the concept of genotype-guided nutritional strategies, where adequate choline intake could modulate SLC44A2 expression and activity. Such personalized dietary interventions may represent a promising adjunct approach for reducing inflammation, improving vascular and joint health, and lowering disease risk in genetically predisposed individuals.

### 5.4. Small Integrin-Binding LIgand, N-Linked Glycoproteins Family (SIBLING)

This protein family comprises a group of molecules closely associated with bone mineralization and skeletal homeostasis. It includes osteopontin (OPN), also known as secreted phosphoprotein 1 (SPP1), integrin-binding sialoprotein (IBSP), dentin matrix protein 1 (DMP1), dentin sialophosphoprotein (DSPP), and matrix extracellular phosphoglycoprotein (MEPE) [117]. These proteins play coordinated roles in regulating extracellular matrix organization, phosphate handling, and mineral deposition in bone and cartilage.

Among them, MEPE has been specifically implicated in erosive hand OA. MEPE is known to regulate phosphate metabolism and bone mineralization, processes that are critical for maintaining skeletal integrity and joint structure [118]. Alterations in these pathways may contribute to the abnormal bone remodeling observed in erosive forms of OA. The involvement of MEPE and related mineralization-associated proteins highlights the relevance of dietary micronutrients, particularly minerals, in OA progression. Evidence supports the importance of balanced mineral intake obtained from whole-food sources to maintain proper bone and cartilage metabolism, rather than excessive or isolated supplementation [119]. This underscores nutrition as a modifiable factor acting on biologically defined pathways relevant to OA.

Importantly, genetic variability further modulates disease risk. The rs17013495 polymorphism in the SPP1 gene has been associated with an increased susceptibility to erosive hand OA [31]. Given the role of SPP1 in bone remodeling and inflammatory signaling, this genetic variant may influence individual responses to mineral availability and skeletal stress.

Overall, these findings emphasize that polymorphisms in genes involved in bone mineralization, such as SPP1, contribute to OA heterogeneity and may interact with dietary mineral status. This supports the rationale for genetically informed nutritional strategies aimed at maintaining mineral balance and skeletal health in patients at increased risk of erosive OA.

### 5.5. M-Phase Phosphoprotein 9 (MPHOSPH9)

MPHOSPH9 undergoes phosphorylation by TTBK2 and is subsequently targeted for degradation via the ubiquitin–proteasome system. This process is dependent on phosphorus availability, as phosphorylation reactions require inorganic phosphate (Pi), an essential mineral involved in numerous cellular functions [120]. Phosphorus intake is therefore tightly regulated, given its fundamental role in energy metabolism, signal transduction, and skeletal homeostasis [121].

Dietary phosphate absorption occurs in the intestine through two mechanisms: an active, carrier-mediated pathway that is partially regulated, and a passive pathway that depends largely on phosphate bioavailability. These processes are integrated within a complex endocrine network that coordinates intestinal absorption, renal excretion, and bone turnover to maintain phosphate homeostasis during conditions of hypo- or hyperphosphatemia [122].

In the context of OA, excessive dietary phosphorus intake has been associated with adverse skeletal and joint outcomes, supporting dietary recommendations that limit phosphorus-rich foods in OA patients. Importantly, genetic factors further influence disease susceptibility. Carriers of the rs7533350451 polymorphism in the MPHOSPH9 gene show an increased risk of developing hip OA [33], suggesting that altered regulation of MPHOSPH9 activity may contribute to joint degeneration. Collectively, these findings highlight the relevance of MPHOSPH9 genetic variation in OA risk and underscore the importance of controlled phosphorus intake. This supports a personalized nutritional approach in which dietary phosphorus is carefully managed, particularly in individuals genetically predisposed to OA, to maintain mineral balance and limit pro-inflammatory signaling.

### 5.6. Ferritin Heavy Chain 1 (FTH1)

The protein FTH1 is involved in adipose tissue inflammation. High serum ferritin levels are associated with obesity and metabolic syndrome, partly due to chronic inflammation [123]. Foods indirectly affect this gene through miRNA 335, an adipogenesis-related microRNA that promotes ferroptosis by targeting FTH1 [124,125]. Furthermore, in bone cells, low vitamin C levels and high glucose conditions affect miRNA-335-5p expression. These two conditions may represent a risk for developing OA. In particular, multiple polymorphisms of the gene coding for FTH1, 133 variants, have been linked to hand OA [102].

## 6. Conclusions

Osteoarthritis (OA) is a complex and heterogeneous disease whose onset and progression are shaped by the interaction of mechanical, metabolic, inflammatory, and genetic factors. Accumulating evidence indicates that diet represents a key modifiable determinant in both the prevention and management of OA, acting not only through biomechanical effects related to body weight but also through direct modulation of inflammatory pathways, cartilage metabolism, and gene expression. This review highlights that macro- and micronutrients, as well as bioactive food-derived compounds, can exert either protective or detrimental effects on joint health depending on their quality, quantity, and overall dietary context. While several nutrients and nutraceuticals have demonstrated beneficial effects on cartilage homeostasis and inflammation, excessive or imbalanced intake of specific dietary components may contribute to metabolic dysfunction and disease progression. These divergent effects underscore the importance of considering diet as a complex pattern rather than as isolated nutrients.

Importantly, growing evidence supports the concept that individual genetic variability significantly influences the biological response to dietary interventions. Common genetic polymorphisms in genes involved in inflammation, cartilage remodeling, mineral metabolism, and metabolic regulation may modify nutrient absorption, bioavailability, and molecular efficacy. This genetic heterogeneity likely contributes to the inconsistent outcomes reported across nutritional studies in OA and highlights the limitations of “one-size-fits-all” dietary recommendations. By integrating insights from nutrigenomics and nutrigenetics, personalized nutrition emerges as a promising strategy to optimize dietary interventions in OA. Tailoring nutritional approaches according to an individual’s genetic background, metabolic profile, and comorbidities may enhance therapeutic efficacy, improve symptom management, and potentially slow disease progression. Although further well-designed longitudinal and interventional studies are needed to validate genotype-based dietary recommendations, the evidence summarized in this review provides a strong rationale for incorporating genetic information into future nutritional guidelines for OA.

In conclusion, a precision nutrition approach that integrates dietary patterns, genetic variability, and metabolic health holds considerable potential to improve the prevention and management of OA, moving toward more individualized and effective strategies in clinical practice.

## Figures and Tables

**Table 1:** Gene polymorphisms in OA and high-sugar diet.

Gene	Polymorphisms	OA Joints	References
HMGN1	rs9981884	Knee and hip	[29]
PAPP-A	rs1321917	Hip	[30]
BMP-6	rs11243284; rs270417	Hand and hip	[31]
CEMIP	rs117564279	Hip	[32]
TACC3	rs7680647; rs4865462	Knee	[22]
USP8	rs4380013	Various joint sites	[33]

**Table 2:** Gene polymorphisms in OA and high-fat diet.

Gene	Polymorphisms	OA Joints	References
COLGALT2	rs11583641; rs1046934; rs12047271; rs1327123	Hip	[68]
MALAT1 (lncRNA)	rs10896015	Hip	[69]
OPN1SW	rs62479589	Hip	[32]
RBM5	rs62262139	Various joint sites	[22]
SPRY4	rs10062749	Hand	[30,33]
	rs10038860	Knee	

**Table 3:** Gene polymorphisms in OA and altered micronutrient diet.

Gene	Polymorphisms	OA Joints	References
CDK2AP1	rs1060105	Knee	[100]
SCAMP2	rs35206230	Knee and hip	[101]
SLC44A2	rs10405617	Various joint sites	[30]
SIBLING	rs17013495	Hand	[31]
MPHOSPH9	rs7533350451	Hip	[33]
FTH1	multiple	Hand	[102]

## Data Availability

No new data were created in this study.
